# Early childhood development and its association with maternal parity

**DOI:** 10.1111/cch.13011

**Published:** 2022-04-09

**Authors:** M Mofizul Islam, Md Nuruzzaman Khan

**Affiliations:** ^1^ Department of Public Health La Trobe University Melbourne Victoria; ^2^ Department of Population Sciences Jatiya Kabi Kazi Nazrul Islam University Mymensingh Bangladesh

**Keywords:** child development, cognitive ability, delayed language, developmental delay, emotional well‐being, physical health

## Abstract

**Background:**

Maternal parity, which is usually measured as the number of children born to a mother, has a substantial impact on the social and environmental factors around children and their development. This paper estimates the Early Childhood Development Index (ECDI) of 3‐ and 4‐year‐old children in Bangladesh and examines the relationship between maternal parity and early childhood development.

**Methods:**

The study analysed nationally representative data from the Bangladesh Multiple Indicator Cluster Survey 2019. The dataset had 9453 children aged from 36 to 59 months. The ECDI was computed following the UNICEF's approach involving psychometric computation of four domains of development: physical, literacy‐numeracy, learning and social–emotional. Since the dataset has a hierarchical structure, we used multilevel logistic regression.

**Results:**

A quarter (25%) of the children were not on track in their early childhood development. Seventy‐one percent were not developmentally on track in the literacy‐numeracy domain, 27% were not in the social–emotional and smaller percentages were not in learning (9%) and physical (1%) domains. There was a significant negative association between maternal parity and ECDI (adjusted odds ratio [AOR] 0.95; 95% CI: 0.91–0.99). Attendance at early childhood education programmes was significantly associated with early childhood development (AOR 1.73; 95% CI: 1.47–2.03). Also, female children, those who were not stunted, located in rural areas, received parental stimulation activities, lived in relatively wealthy households or had mothers who had received secondary or further education were more likely than others to be on track of early childhood development.

**Conclusions:**

Early childhood development is negatively correlated with maternal parity. The literacy‐numeracy domain constitutes the major developmental delay. Programmes for parental awareness should be widely expanded.

**Key messages**

- A quarter (25%) of the children aged 36 to 59 months were not on track in their early childhood development.
- The literacy‐numeracy domain constitutes the major developmental delay.
- This study found a significant negative association between maternal parity and early childhood development.
- Early childhood education programmes, education for girls and the creation of parental awareness should be widely expanded.

## 1 INTRODUCTION

There is growing evidence that early childhood is the most important developmental phase throughout the lifespan. Healthy early child development, which includes the physical, social–emotional and language‐cognitive domains of development, influences children's overall health and well‐being and establishes their developmental trajectories and life‐courses (Loizillon et al., 2017; UNICEF, WHO, & World Bank, 2016). It is now well accepted that apart from biological factors, children's development is shaped by the quality of their homes, neighbourhood environments, characteristics of their parents (Downey, 2001) and social factors (Ranjitkar et al., 2019). This growing evidence has prompted national and international calls for investment in early childhood development (Wilkinson, Marmot, & WHO, 1998) and for researchers to identify specific factors in children's social environments that facilitate or hinder early childhood development and can be modified. However, the evidence so far for the effects of social factors is mostly context‐specific and varies between studies and settings (Donald et al., 2019; McDonald et al., 2016; Walker et al., 2011).

In the research undertaken to identify significant factors associated with early childhood development, maternal parity has received little attention. Maternal parity, which is usually measured as the number of children born to a mother, has a substantial impact on the social and environmental factors around children and their development because the family system and structure vary between one‐child and multichild families and keep changing with each new birth (Fish & Stifter, 1993). In earlier literature, the effects of sibship sizes (i.e., number of children in the family) on intellectual skills or educational achievement received the most attention (Downey, 2001; Guo & VanWey, 1999; Lu & Treiman, 2008), while aspects such as social, emotional and physical development received inadequate attention. In recent literature, quantitative studies investigating factors that influence early childhood development were sometimes adjusted for maternal parity, but it was seldom the main focus (de la Rochebrochard & Joshi, 2013). Moreover, although a small number of studies identified maternal parity as important, the findings are inconsistent (Aronen, 2009; Hayashida & Nakatsuka, 2014). Also, the ways that the studies calculated early childhood development varied, and the developmental indicators were rarely validated appropriately, making comparisons of the findings impossible. In fact, the dearth of globally accepted indicators for child development has hampered the progress of research in this area (Loizillon et al., 2017).

During 2003–2009, UNICEF developed and validated the Early Childhood Development Index (ECDI), which measures four areas of development: physical, literacy‐numeracy, learning and social–emotional. The resulting scores reflect whether children are developmentally on track in each of the four domains and overall. The ECDI reflects the population‐based normative distribution of developmental status (Loizillon et al., 2017). This index is the first international population‐based measure of early childhood development for low‐ and middle‐income countries. Using population‐representative data from the Multiple Indicator Cluster Survey (MICS) programme, this paper estimates the number of 3‐ and 4‐year‐old children who were on track in their development and the relationship between maternal parity and early childhood development of their children in Bangladesh.

## 2 MATERIAL AND METHODS

### 2.1 Data

We used the 2019 MICS data for Bangladesh. MICS is a nationally representative and internationally standardized household survey developed by UNICEF that captures information about children in low‐ and middle‐income countries (Khan & Hancioglu, 2019; UNICEF, 2021). A two‐stage, stratified cluster sampling was used for data collection. The urban and rural areas within each of 64 administrative districts were identified as the main sampling strata. First, within each stratum, a specified number of primary sampling units were selected systematically with probability proportional to size. Second, after identifying potential households within the selected enumeration areas, a systematic sample of 20 households was drawn from each primary sampling unit (Bangladesh Bureau of Statistics & UNICEF‐Bangladesh, 2019). A total of 61 242 households were included in the survey. Mothers or caretakers of children under‐5 were asked a series of questions including early childhood developmental aspects. For the current study, relevant data for under‐5 children, their parents and households were extracted. Information on early childhood development was available for 9453 children aged 36 to 59 months who constituted the sample for this study.

### 2.2 Exposure variable and other covariates

Maternal parity was the exposure variable. Other covariates included the children's age, sex, nutritional status measured through stunting, attendance at early childhood education programmes, mothers' educational status, places of residence, households' wealth status and the parental stimulation activities they had. The structural covariates were districts, communities and households. The primary sampling units were considered to be their communities.

The standards of the WHO were used for identifying children with stunting. Hence, children whose height‐for‐age was more than 2 standard deviations below the median of the reference population were considered short for their age and are classified as stunted (WHO, 2021). Parental stimulation was measured by asking the primary caregivers whether any household members had engaged in the following six activities with the children in the past 3 days: reading books or looking at pictures, telling stories, singing songs, taking the child outside, playing with the child and naming, counting or drawing together. A summary score was created, which varies from 0 (no paternal engagement in any stimulation activity) to 6 (paternal engagement in all stimulation activities). This approach has been used as a measure of caregiving and stimulation in several previous studies (Bornstein et al., 2015; Bornstein & Putnick, 2012; Jeong et al., 2016; Sun et al., 2016).

### 2.3 Outcome variable

In the MICS dataset, information for the ECDI was collected for 3‐ and 4‐year‐old children. We computed the ECDI for following the UNICEF's approach involving psychometric computation, the details of which can be sourced elsewhere (Loizillon et al., 2017). Briefly, this index includes 10 questions covering four domains of early childhood development: language‐cognitive, physical, social–emotional and approaches to learning (Table 1). Children are regarded as having healthy early childhood development if they are developmentally on track in at least three of these domains.

**TABLE 1:** Four domains and their questions, responses and assessment of Early Childhood Development Index

Four domains	10 questions	Question item on track if the answer is …	Domain developmentally on track if …
Literacy‐numeracy	1. Can the child identify or name at least 10 letters of the alphabet?	Yes	At least two items on track
	2. Can the child read at least four simple, popular words?	Yes	
	3. Does the child know the name and recognize the symbol of all numbers from 1 to 10?	Yes	
Physical	4. Can the child pick up a small object with two fingers, like a stick or a rock from the ground?	Yes	At least one item on track
	5. Is the child sometimes too sick to play?	No	
Learning	6. Does the child follow simple directions on how to do something correctly?	Yes	At least one item on track
	7. When given something to do, is the child able to do it independently?	Yes	
Social–emotional	8. Does the child get along well with other children?	Yes	At least two items on track
	9. Does the child kick, bite, or hit other children or adults?	No	
	10. Does the child get distracted easily?	No	

*Note*: Percentage of children age 3–4 years (36–59 months) who are developmentally on track in at least three of the above four domains.

Abbreviation: ECDI, Early Childhood Development Index.

*Source*: UNICEF, Multiple Indicator Cluster Surveys 6 questionnaires and indicators, http://mics.unicef.org /, 2018.

### 2.4 Data analysis

We conducted descriptive analysis to summarize the characteristics of the children and their ECDI status, attendance at early childhood education programmes, nutritional status, households' economic status, places of residence and mothers' educational status. The dataset has a hierarchical structure: children are nested in households, households are nested in primary sampling units (i.e., communities) and these are then nested in the 64 administrative districts. Thus, we used multilevel logistic regression—both for overall ECDI and the four individual domains as outcome variables. We used a three‐level multilevel model: children (level 1), community (level 2) and districts (level 3). The main study factor, maternal parity, was included as a continuous variable in the regression models. Three models were developed. Model 1 was the null model and did not have any predictor variables, Model 2 examined the relationship between maternal parity and ECDI of their children and Model 3 was the final model that examined the relationship while being adjusted for other covariates.

The general contextual effects have been estimated using the between‐neighbourhood variance and the median odds ratio (Larsen & Merlo, 2005; Merlo et al., 2016). The median odds ratio translates the area level variance in the odds ratio scale and quantifies the variation between clusters by comparing two individuals from two randomly chosen different clusters. In simple terms, the higher the median odds ratio, the greater the general contextual effect. Survey weights were applied when tabulating the descriptive statistics but not in the multilevel regressions. We used Stata for statistical analysis and Microsoft Excel for graphical presentation. The associations between maternal parity and ECDIs were presented as odds ratios (OR) with 95% confidence intervals (CIs).

## 3 RESULTS

The dataset had complete ECDI for 9380 children aged from 36 to 59 months. These children lived in 9209 households, enumerated from 3220 primary sampling units. On average, their mothers had given birth to 2.37 children (SD ± 1.28). A quarter (25%) of the children were not on track in their ECDIs. Only 19% had ever attended early childhood education programmes. Table 2 presents children's sociodemographic characteristics and their ECDIs. Age‐wise segregation shows that 84% of those who attended early childhood education programmes were 4‐year‐old children (not shown in the table).

**TABLE 2:** Children's sociodemographic characteristics and their Early Childhood Development Index

Variables	Overall %	ECDI on track %^a^
Children who are developmentally on track		
Early child development index (ECDI)	74.9	100.0
Literacy‐numeracy	28.8	98.8
Physical	98.6	75.7
Socio‐emotional	72.9	92.6
Learning	91.4	81.0
Maternal parity		
1	24.8	77.0
2	38.7	75.5
3	21.2	75.2
4	9.1	70.4
5	3.4	68.4
6	1.6	61.8
7 or more	1.2	64.5
Children's age in months		
36–47	51.0	68.7
48–59	49.0	81.2
Children's sex		
Male	51.8	71.5
Female	48.2	78.4
Nutritional status of children		
Stunted	28.1	70.3
Not stunted	71.9	76.8
Child ever attended early childhood education programme		
Yes	19.4	86.0
No	80.6	72.2
Number of parental stimulation activities in last 3 days		
0	13.2	70.1
1	6.9	67.4
2	8.7	73.6
3	9.5	75.7
4	11.9	73.5
5	13.3	77.3
6	36.5	77.7
Place of residence		
Urban	21.0	78.2
Rural	79.0	74.0
Mothers' education status		
None or per‐primary	13.1	68.6
Primary	24.4	69.3
Secondary	48.1	76.9
Higher secondary or more	14.4	83.2
Household's wealth quintile		
Poorest	22.3	68.3
Poorer	19.9	71.5
Middle	18.7	75.4
Richer	19.3	75.9
Richest	19.8	84.1

*Note*: All percentages are weighted.

^a^ Among the children with attributes described in the first column, for example, 51.8% were male children and among them 71.5% were on track of their ECDI.

**FIGURE 1:**
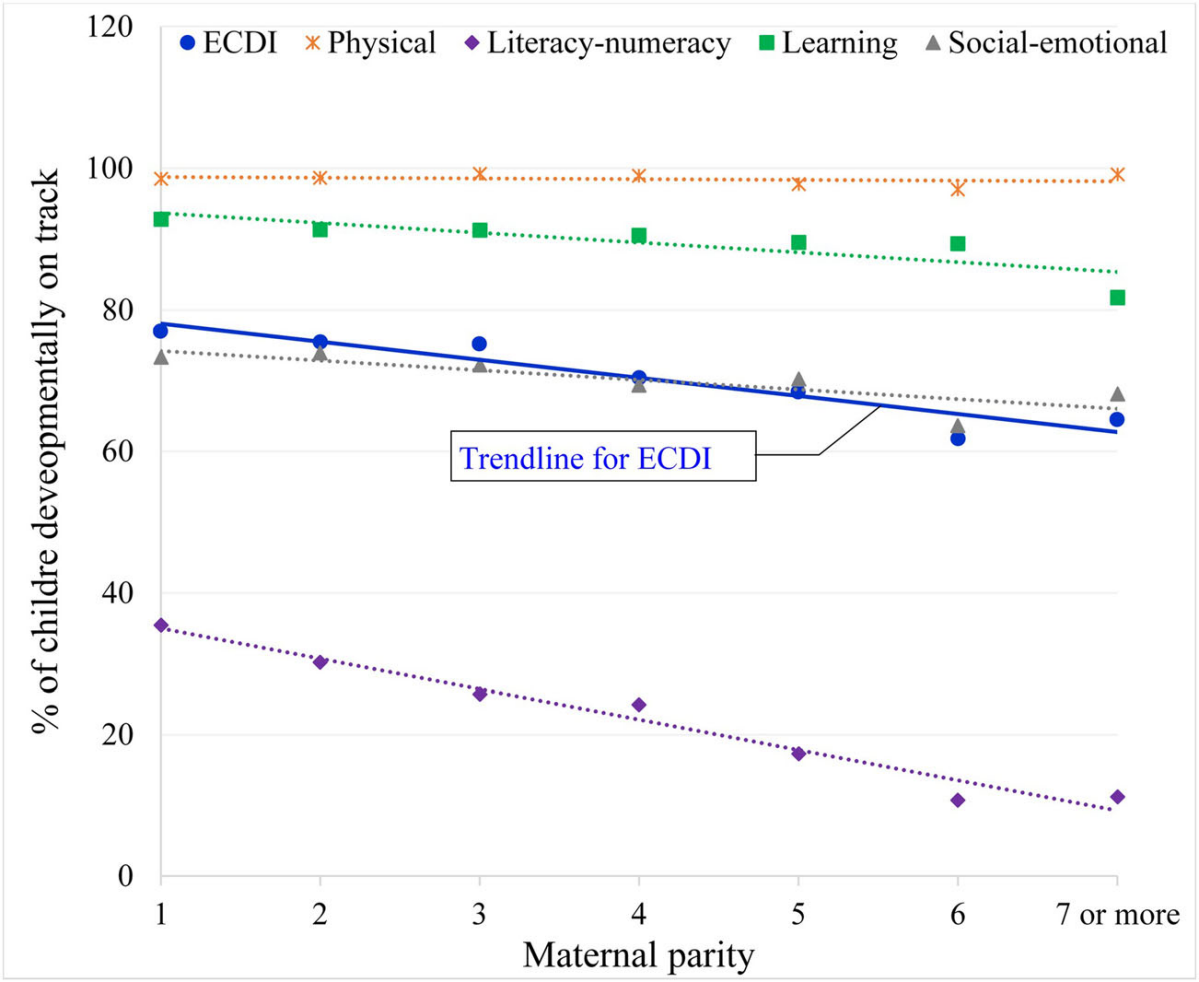
Univariable relationship between number of children ever born and Early Childhood Development Indexes. *Note*: ECDI: Early Childhood Development Index

Univariate analysis with the ECDI and maternal parity shows a significant negative association. Figure 1 shows the scatterplot of maternal parity and the ECDI along with its four domains among children aged 3 and 4 years. The trendlines of this picture show an inverse relationship between four domains of the ECDI and maternal parity. The scatterplot also indicates that a substantial proportion (71%) of children was not developmentally on track in the literacy‐numeracy domain, and it was the worst‐performing index of all. Children who attended early childhood education programmes were more likely than those who did not to be developmentally on track in the literacy‐numeracy domain. The social–emotional domain was the second most challenging one, followed by the learning domain and then the physical domain.

Table 3 presents the results of the multilevel logistic regression. Model 1 is the intercept‐only empty model with no predictors. The main exposure factor, maternal parity, was included in Model 2 with no other covariates. Model 3 is the complete model with all covariates included. The *p*‐values of the likelihood ratio test were lower than 0.001 for all of these models, suggesting that the multilevel model is preferable to logistic regression. The random‐effects variance and median odds ratio indicate that the district‐level context has a substantial effect and is important for understanding children's ECDIs.

**TABLE 3:** Multilevel logistic regression examining the association between maternal parity and Early Childhood Development Index in Bangladesh, *n* = 8811

Variable	AOR (95% CI)		
	Model 1	Model 2	Model 3
Maternal parity		0.91 (0.87–0.94)	0.95 (0.91–0.99)
Child's age			
3 years (reference)			1
4 years			2.00 (1.79–2.23)
Child's sex			
Male (reference)			1
Female			1.42 (1.28–1.58)
Stunted			
No (reference)			1
Yes			0.89 (0.80–0.99)
Child ever attended early childhood education programme			
No (reference)			1
Yes			1.73 (1.47–2.03)
Parental stimulation in last 3 days			1.03 (1.01–1.06)
Mother's education level			
None or per‐primary (reference)			1
Primary			0.98 (0.83–1.17)
Secondary			1.23 (1.03–1.47)
Higher secondary or more			1.52 (1.20–1.94)
Wealth quintile			
Poorest (reference)			1
Poorer			1.03 (0.88–1.19)
Middle			1.10 (0.93–1.29)
Richer			1.10 (0.92–1.31)
Richest			1.63 (1.31–2.03)
Place of residence			
Urban (reference)			1
Rural			1.18 (1.02–1.37)
Constant	2.96 (2.53–3.46)	3.72 (3.10–4.45)	1.19 (0.87–1.63)
*p* for LR test versus logistic model chi^2^	<0.001	<0.001	<0.001
Random effect variance			
District level	0.35	0.35	0.38
Community level	0.05	0.04	0.03
Median odds ratio (95% CI)			
District level	1.77 (1.57–1.96)	1.76 (1.56–1.96)	1.80 (1.59–2.01)
Community level	1.23 (0.98–1.49)	1.22 (0.95–1.48)	1.18 (0.86–1.51)

Abbreviations: AOR, adjusted odds ratio; CI, confidence interval.

The constant value of the intercept‐only model (i.e., Model 1) is the estimated odds of early childhood development being on track for the children. The adjusted odds ratio (AOR) of Model 2 was 0.91 (95%CI: 0.87–0.94), which shows that maternal parity has a significant negative effect on the ECDI. The AOR in Model 3 shows that maternal parity is a significant factor in the ECDI. For every additional child, the odds ratio for a child being developmentally on track is reduced by 5%. In the final model, several other variables were found to have significant effects on children's ECDIs. Four‐year‐old children were significantly more likely than 3‐year‐old children to be on track in their early childhood development. Furthermore, female children (AOR 1.42, 95%CI 1.28–1.57) and those who had attended early childhood education programmes (AOR 1.73; 95%CI 1.47–2.03) were more likely than others to be developmentally on track. The higher the scores for parental stimulation activities in the previous 3 days, the more likely it was that the children were on track in their ECDIs (AOR 1.03; 95%CI 1.01–1.06). Children whose mothers had completed higher secondary or further education were also significantly more likely to be developmentally on track. There was a somewhat positive trend regarding household wealth status in terms of the ECDI, with AOR increasing gradually for the higher levels of wealth quintiles; however, the association was statistically significant only for children from the most affluent households. In addition, children living in rural areas were more likely to be developmentally on track than those in urban areas (AOR 1.18; 95%CI 1.02–1.37).

Multilevel regression with the individual domains of the ECDI shows that the literacy‐numeracy and social–emotional domains were significantly negatively associated with higher maternal parity (Table 4). Of these domains, the AOR for the literacy‐numeracy domain was substantially affected by the numbers of siblings. The relationships of other covariates continue to be similar to those shown in Model 3.

**TABLE 4:** Multilevel logistic regression examining the association between maternal parity and four domains of Early Childhood Development Indexes

ECDI domains	AOR (95% CI)
ECDI	0.95 (0.91–0.99)
Physical	1.06 (0.90–1.25)
Literacy‐numeracy	0.89 (0.85–0.94)
Learning	0.98 (0.92–1.05)
Social–emotional	0.95 (0.91–0.99)

*Note*: Each model was adjusted for all covariates.

Abbreviations: AOR, adjusted odds ratio; CI, confidence interval; ECDI, Early Childhood Development Index.

## 4 DISCUSSION

The findings of this study suggest that, overall, a quarter of the children were not on track in their early childhood development, which is much higher than is estimated in many other low‐ and middle‐income countries (Emerson et al., 2018). The performance in literacy‐numeracy was the worst of the four domains of the ECDI. The findings also suggest that children with relatively large sibship sizes are significantly more likely not to be on track in their early childhood development. The other important finding is that female children who were not stunted, had attended early childhood education programmes, received parental stimulation or lived in relatively wealthy households or whose mothers had received secondary or further education were more likely than their counterparts, respectively, to be on track in their early childhood development.

Our results suggest that literacy‐numeracy is the most challenging domain, which substantially delay children's development. McCoy et al. (2016) argue that the literacy‐numeracy items (recognizing words, letters and numbers) are too difficult for 3‐ and 4‐year‐old children. Gil et al. (2020) argue that the literacy‐numeracy domain indicators are related to training and opportunities in early schooling. Our results agree with this. However, sadly, attendance at early childhood education programmes is relatively low (<20%) in Bangladesh. In 2010, Bangladesh formally recognized preprimary as the first stage of the education system and established several programmes to initially include all 5‐year‐old children and then gradually the 4‐year‐old children too (Government of Bangladesh, 2010). Nearly 100% of government primary schools now offer 1 year of preprimary education at no cost (Bangladesh Bureau of Statistics & UNICEF‐Bangladesh, 2019). Many private kindergartens, madrasahs and nongovernmental organizations also offer preprimary education. However, the relatively low rate of attendance (<20%) could be because the programmes do not yet include all children aged 3 and 4 years (Tribune Desk, 2020). The attendance at early childhood education programmes can accrue additional developmental benefits, as they also offer most of the parental stimulating activities such as reading books, telling stories, singing songs and naming, counting or drawing with the child.

Several covariates in our study, including stunting, access to early childhood education, the household's wealth status, the mother's educational status and the location of the residence, have been consistently identified as important for early childhood development (Frongillo et al., 2017; Kang et al., 2018; Sk et al., 2020). These are indeed a set of social determinants of health (Islam, 2019) and highlight that early childhood development follows a social gradient. Unfavourable social determinants of health increase exposure among young children to biological and psychosocial risks that subsequently affect development (Amugsi et al., 2020; Walker et al., 2007). In wealthy countries, maternal parity may not have a significant impact on early childhood development because the negative effects of high parity can be offset by the positive effects of factors such as good nutrition, educated mothers, healthy environments and access to early childhood education. However, in resource‐poor settings, relatively large sibship sizes can impact children's shares of base resources such as food, clothes and shelter, and they may only receive minimal supervision (Downey, 2001). As the risks accumulate, development may be compromised in a compound manner (Moore et al., 2015; Walker et al., 2007).

While improving social conditions in low‐income countries takes time, at least some programmes can be established for parents from relatively low socioeconomic backgrounds, as they may not have the same access to health information as other families (Begum, 2019). Parental knowledge of childhood development can affect attitudes and behaviours and impact their children's outcomes through a “mechanism of change” (Moran et al., 2004). Parenting programmes can target educating parents in families of lower socioeconomic status about the importance of early childhood development, their engagement, child nutrition and early childhood learning (Aboud, 2007). While the Comprehensive Early Childhood Care and Development Policy 2013 formulated by the government provide an operational framework for developing comprehensive programmes for children (Ministry of Women and Child Affairs, 2013), ensuring its proper implementation needs proactive measures.

There could be several explanations for the negative association that was observed between the maternal parity and the ECDI. Since we had no information about the genetics of the children, and we examined only the psychosocial factors, one possible explanation is that of “resource dilution” (Downey, 2001), which refers to a lower parental investment in children that goes along with increasing parity and/or competition between siblings for finite resources (Helfrecht & Meehan, 2016). A second explanation is the omitted variable bias. There may have some unmeasured confounding variables that were not split equally between the children who were developmentally on track and those who were not, thus producing a spurious association. Nevertheless, there is a high chance that such an unequal distribution is affected by resource dilution. Another explanation could be that the fertility rate is relatively low for families that can afford multiple children, while the rate remains higher among the least affluent families (Bangladesh Bureau of Statistics, 2015). However, the findings of this study warrant due attention, particularly in countries like Bangladesh, which has a large population in a relatively small piece of land: 166 million in 148 460 km^2^. We recommend further research to find causes for the association that was observed in our study. The expected benefit that would come from further research is important for Bangladesh and for similar countries.

An important finding of the multilevel regression is that the developmental scores of children living in rural areas were better than those in urban areas. This observation is quite the opposite of what has usually been observed in other settings (Gil et al., 2020). There could be several reasons for this finding. First, more of the children in urban areas who were developmentally on track had also attended early childhood education programmes than the children in rural areas who were on track, and after adjustment in regression for early childhood education, this offered a net performance that was better for rural children. Second, this finding may be partly attributable to measurement bias; since many mothers in rural areas had only preprimary or even less education, some may have erroneously identified their children as being on track in the literacy‐numeracy domain and therefore contributed to differential misclassification.

## 5 LIMITATIONS

This study has several limitations. Firstly, this is a cross‐sectional study. Thus, the relationship between maternal parity and early childhood development is associational only. Secondly, although we adjusted the regression models for many potential confounders, it is not unlikely that some residual confoundings remain unadjusted. Future research should examine this relationship using a prospective cohort dataset. Lastly, the contemporary theoretical conceptualization of child development incorporates the biopsychosocial model of health, which puts importance on both nature and nurture. We were unable to account for the biological and genetic endowment of the children.

## 6 CONCLUSION

Our study found a negative association between maternal parity and early childhood development. Among the four domains of early childhood development, the performance in literacy‐numeracy was the worst. Several factors such as children's nutritional status, their attendance at early childhood education programmes, the parental stimulation activities they had, their mothers' education and their households' wealth status were significantly associated with early childhood development, and this highlights the importance of social determinants of health. While addressing all these factors may not be an achievable goal over the short term, particularly within the context of a developing country, ongoing efforts for early childhood education, education for girls and the creation of parental awareness should be widely expanded.

## AUTHOR CONTRIBUTIONS

Conceptualization: MMI; formal analysis: MMI; visualization: MMI; writing ‐ original draft: MMI; critical review: MMI, MNK.

## Data Availability

The data that support the findings of this study are publicly available on the website of UNICEF MICS upon its approval.
